# CCR9 Promotes Migration and Invasion of Lung Adenocarcinoma Cancer Stem Cells

**DOI:** 10.7150/ijms.40864

**Published:** 2020-03-26

**Authors:** Lin Lu, Huan Du, Haowei Huang, Chenxi Wang, Peipei Wang, Zhiqiang Zha, Yong Wu, Xia Liu, Chengyin Weng, Xisheng Fang, Baoxiu Li, Haibo Mao, Lina Wang, Mingmei Guan, Guolong Liu

**Affiliations:** 1 Department of Medical Oncology, Guangzhou First People's Hospital, Guangzhou Medical University, Guangzhou, Guangdong, China, 510180; 2 Department of Medical Oncology, Guangzhou First People's Hospital, School of Medicine, South China University of Technology, Guangzhou, Guangdong, China

**Keywords:** CCR9, lung adenocarcinoma, cancer stem cells, migration, invasion

## Abstract

**Aim**: CC chemokine receptor 9 (CCR9) interacts with its exclusive ligand CCL25, resulting in promoting tumor progression and metastasis. However, the effect and mechanisms of CCR9 on lung adenocarcinoma distant metastasis remain largely unknown. To preliminary clarify the underlying mechanisms, we investigate the correlation between CCR9 and ALDH1A1^+^cancer stem cells (CSCs), as well as the effect of CCR9 on the migration and invasion of CSCs.

**Methods**: Immunohistochemistry was performed to detect the expression of CCR9 in lung adenocarcinoma tissues. The correlations of CCR9 with distant metastasis and overall survival were investigated. Serial paraffin-embedded tissue blocks were used to detect ALDH1A1^+^CSCs expression. The correlations between CCR9 expression and ALDH1A1^+^CSCs were evaluated. We further studied the effect of CCR9/CCL25 on the migration and invasion of CSCs using transwell assays.

**Results**: There were positive correlations between CCR9 expression and distant metastasis, as well as poor overall survival. Patients with high CCR9 expression were more likely to develop distant metastasis and demonstrated poorer overall survival than patients with low CCR9 expression. In addition, there was positive correlation between the expression of CCR9 and ALDH1A1 in the same tumor microenvironment. ALDH^high^ CSCs demonstrated enhanced expression of CCR9 than ALDH^low^ cells. Further transwell assays demonstrated that the numbers of CSCs migrated or invaded in response to CCL25 were more than that without CCL25 stimulation. Additional application of anti-CCR9 antibody reversed the CCL25-induced migration and invasion of CSCs.

**Conclusions**: In summary, our study demonstrated that CCR9/CCL25 promoted the migration and invasion of CSCs, which might contribute to distant metastasis and poor overall survival. Our findings provided evidence that CCR9/CCL25 could be used as novel therapeutic targets for lung adenocarcinoma.

## Introduction

Lung cancer is the most common cancer worldwide, and its morbidity and mortality rank first among all cancers 1. Non-small cell lung cancer (NSCLC) accounts for about 83% of lung cancer. As the largest subtype of NSCLC, the incidence of lung adenocarcinoma is increasing yearly 2. Besides traditional anticancer strategies, the advancements of the targeted therapies and immune checkpoint inhibitors benefit more lung adenocarcinoma patients 3. However, most of the patients were diagnosed at an advanced stage and with a high possibility to develop distant metastasis. The 5-year survival rate for lung adenocarcinoma remains unsatisfactory 4. Thus, it's of great importance to find molecular biomarkers which could predict metastasis and prognosis.

Chemokine/chemokine receptor axis plays important roles in determining tumor development and metastasis 5-7. Chemokine receptors were expressed on both immune cells and tumor cells, which could recruit immune cells to the tumor microenvironment, as well as driving metastatic process 6, 7. CCR9 was identified as an organ- specific chemokine receptor, which could specifically bind with thymus-expressed chemokine CCL25/ TECK 8. The expression of CCR9 and CCL25 have been identified in some solid tumors, including ovarian cancer 9, breast cancer 10, prostate cancer 11, pancreatic cancer 12 and esophageal cancer 13. Increased CCR9 expression was correlated with poor overall survival 9, 14. CCR9/CCL25 axis played an important role in tumor invasion, migration, drug resistance and apoptosis 15, 16. The application of anti-CCR9 antibody could inhibit the migration and invasion of tumor cells 17. Moreover, application of anti-CCR9 monoclonal antibody demonstrated therapeutic efficacy in leukemia cell xenografts. These studies indicated that CCR9/CCL25 demonstrated great potential in tumor-targeted therapy like other chemokine and chemokine receptors 18, 19.

CCR9 was also detected highly expressed in non- small cell lung cancer (NSCLC) 17. Increased CCR9 expression was correlated with poor overall survival 14. However, little is known about the effect and mechanism of CCR9 on distant metastasis in lung adenocarcinoma. In this study, we found that increased expression of CCR9 was correlated with distant metastasis in lung adenocarcinoma patients. There was positive correlation between the expression of CCR9 and ALDH1A1. Expression level of CCR9 on CSCs was much higher than that on non-CSCs, which resulted in promoting the migration and invasion of CSCs.

## Material and Methods

### Ethical statement

This study was approved by the Ethics Committee of Guangzhou First People's Hospital, and written informed consent was obtained from all patients prior to surgery. All procedures involving human participants were in accordance with the ethical standards of the institutional and*/*or national research committee and with the 1964 Helsinki declaration and its later amendments or comparable ethical standards.

### Patients and specimens

Paraffin-embedded tumor tissues were obtained from 76 cases of lung adenocarcinoma patients who underwent surgery in Guangzhou First People's Hospital between 2007 and 2016. None of the patients had received any anticancer strategies prior to the surgery. All patients were histologically confirmed as lung adenocarcinoma. There were 46 males and 30 females with the average age of 61 years (range from 40 to 78). According to the 8th edition of the International Association for the Study of Lung Cancer (IASLC) published in 2017, there were 43 cases staged as I-II and 33 cases staged as III-IV. The median follow-up period was 60.4 months (range, 1-101 months).

### Culture of tumor cells

Lung adenocarcinoma cell line A549 was purchased from Cellcook Biotech Co.,Ltd (Guangzhou, China) and cultured in complete medium consisting of RMPI 1640 supplemented with 10% heat-inactivated fetal bovine serum, 100 μg/mL streptomycin, 100 U/mL penicillin.

### Immunohistochemistry

The paraffin-embedded sections were cut at a thickness of 2 μm. The immunohistochemical analysis was performed as previously described 20. After being baked at 60 °C for 60 minutes, the sections were dewaxed and rehydrated with xylene and graded alcohol. After antigen retrieval, the sections were incubated overnight at 4°C with primary antibodies as follows: rabbit anti-CCR9 polyclonal antibody (1:200, Abcam), rabbit anti-CCL25 polyclonal antibody (1:200, ABclonal and 1:200, Signalway Antibody), rabbit anti-ALDH1A1 polyclonal antibody (1:400,Abcam). The sections were washed with PBS and developed with diaminobenzidine (DAB). Finally, the sections were counterstained with hematoxylin, dehydrated and then sealed.

### Evaluation of immunohistochemical staining

Five random non-crossover fields were selected under a microscope at 400 times magnification. The average positive percentage of cells in each field was taken as the percentage of positive cells in the section. And the percent scores were defined as follows: 0, ≤ 5%; 1, 5- 25%; 2, 25-50%; 3, > 50%. The staining intensity scores were defined as follows: 0, no staining; 1, weak staining; 2, moderate staining; 3, strong staining. The percentage of positive cells and the staining intensity score were multiplied to obtain a final score. The patients were divided into two groups: low expression group (0 - 4 points) and high expression group (6-9 points) as previously described 21.

### ALDEFLUOR assay

The ALDEFLUOR has been used as a unique marker to isolate cancer stem cells (CSCs) from lung cancer 22. The ALDEFLUOR^+^/ALDH^high^ subpopulation was isolated using ALDEFLUOR kit (Stem Cell Technologies) as previously described 22, 23. Tumor cells stained with ALDH inhibitor diethylaminobenzaldehyde (DEAB) was used as negative control.

### Flow cytometry

ALDH^high^ or ALDH^low^ cells were sorted using flow cytometry. Both ALDH^high^ and ALDH^low^ cells were incubated with PE-conjugated CCR9 antibody. The expression of CCR9 was detected using flow cytometry as previously described 23.

### RNA extraction and real time quantitative PCR (RT-qPCR)

Total RNA from tumor cells was extracted using TRIzol solution and the cDNA was synthesized as previously described 24, 25. The resulting cDNA was performed to RT-qPCR to evaluate the relative mRNA levels of CCR9 and internal control GAPDH using the following premiers: 5'- CGATGAACACGAGCCAGTACAAG-3' (F) and 5'- TGCATCTGACTGACCCACCA-3' (R) for CCR9; 5'-CTCCTCCTGTTCGACAGTCAGC-3' (F) and 5'-CCCAATACGACCAAATCCGTT-3' for GAPDH. The relative mRNA values were calculated using the comparative threshold cycle (2-^ΔΔ^CT) method. And the relative expression levels of CCR9 were normalized to the value of GAPDH. The experiments were repeated at least three times.

### Cell migration and invasion

Cell migration and invasion assays were performed using 24-well plates with 8-μm pore size polycarbonate membrane inserts (Corning, USA). For cell invasion assay, polycarbonate membrane inserts were coated with a thin layer of 0.5 mg/mL Matrigel Basement Membrane Matrix. The lower chambers were filled with 500 μL of complete medium. For some experimental group, CCL25 (100 ng/ml) was added to the lower chamber. For some experimental group, anti-CCR9 (1μ g/ml, R&D) was added to the top chamber. After the cells migrated or invaded to the lower surface, the membrane was stained using crystal violet. The results were calculated by counting the stained cells under an inverted microscope (10 fields per membrane). Each experiment was repeated at least 3 times.

### Statistics

All statistical calculations were performed using software (version 25.0; SPSS Inc., Chicago, IL, USA) or GraphPad Prism 7 (GraphPad software). Student's t-test was used to value the differences between two experiment groups. Chi-square test was used to determine the relationship between CCR9 expression or ALDH1A1 expression with metastasis, and the correlation between CCR9 and ALDH1A1. Survival analysis was performed by the Kaplan-Meier method and log-rank test. Prognostic value of various factors was analyzed by univariate and multivariate analysis (Cox proportional hazards regression model). A two-tailed *P* value < 0.05 was considered statistically significant.

## Results

### Increased CCR9 expression correlated with distant metastasis and poor outcomes of lung adenocarcinoma patients

**Figure 1:**
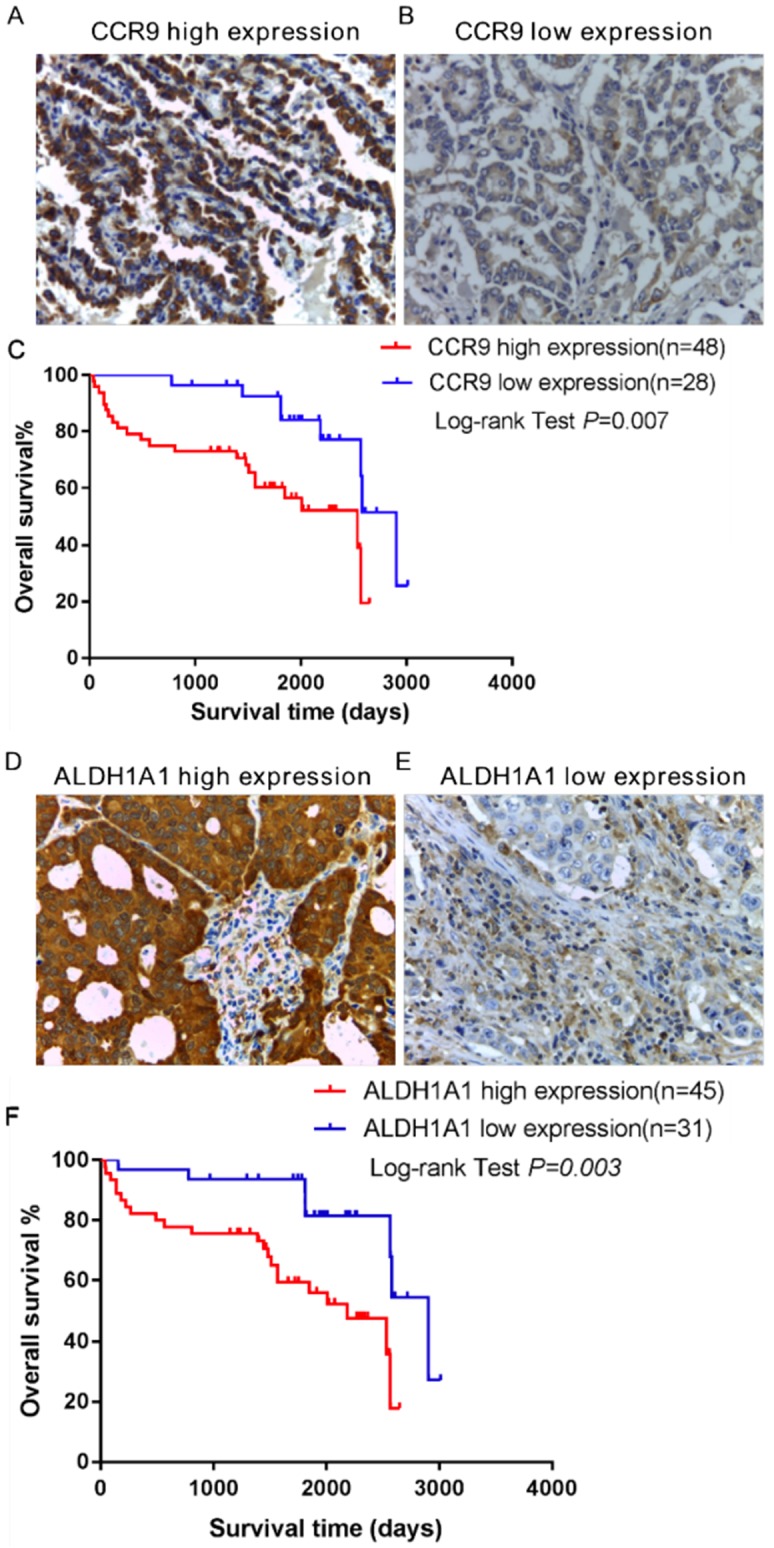
** The expression and prognostic value of CCR9 and ALDH1A1 in lung adenocarcinoma patients.** Representative microphotographs of CCR9 expression: (A) High CCR9 expression; (B) Low CCR9 expression. (C) Kaplan-Meier curve of overall survival predicted by CCR9 expression. Patients with low CCR9 expression demonstrated OS than patients with high CCR9 expression. Representative microphotographs of high ALDH1A1 expression (D) and low ALDH1A1 expression (E). (F) Increased ALDH1A1 expression was positively correlated with poor overall survival. Original magnification: 400×.

The expression of CCR9 and its ligand CCL25 in the tumor tissues of lung adenocarcinoma patients was investigated using immunohistochemistry. High expression of CCR9 was detected in 48 cases, while 28 cases displayed low CCR9 expression. The representative photomicrographs are shown in Figure 1A and B. However, CCL25 expression was barely detected in lung adenocarcinoma tissues (supplementary Figure 1). We further analyzed the relationship between CCR9 and distant metastasis. As shown in Table 1, there was significant correlation between CCR9 expression and distant metastasis (*P* = 0.026). The high expression rates of CCR9 in patients staged as M0 and M1 were 53.8% and 83.3%, respectively, while the low expression rates were 46.3% and 16.7%, respectively.

Kaplan-Meier survival analysis was performed to investigate the association between CCR9 expression and the overall survival (OS) of lung adenocarcinoma patients. As shown in Figure 1C, there was significant correlation between CCR9 expression and the OS (Figure 1C,* P* = 0.007). Patients with high CCR9 expression had poorer OS than those with low CCR9 expression.

### Increased expression of ALDH1A1+ cancer stem cells (CSCs) was correlated with distant metastasis and poor overall survival

Furthermore, the expression of ALDH1A1+ CSCs in the same tumor microenvironment was detected using serial paraffin-embedded sections. ALDH1A1 was highly expressed in 45 cases and lowly expressed in 31 cases. Figure 1D and E showed representative images of ALDH1A1^+^CSCs. Kaplan- Meier survival analysis showed that ALDH1A1 expression could predict the overall survival (OS). Lung adenocarcinoma patients with high ALDH1A1 expression had poorer OS than those with low ALDH1A1 expression (*P* = 0.003, Figure 1F). There was positive correlation between the expression of ALDH1A1^+^CSCs and distant metastasis (Table 2, *P* = 0.016). Patients in the ALDH1A1 high expression group were more likely to develop distant metastasis.

### CCR9 expression was positively correlated with ALDH1A1 expression

**Figure 2:**
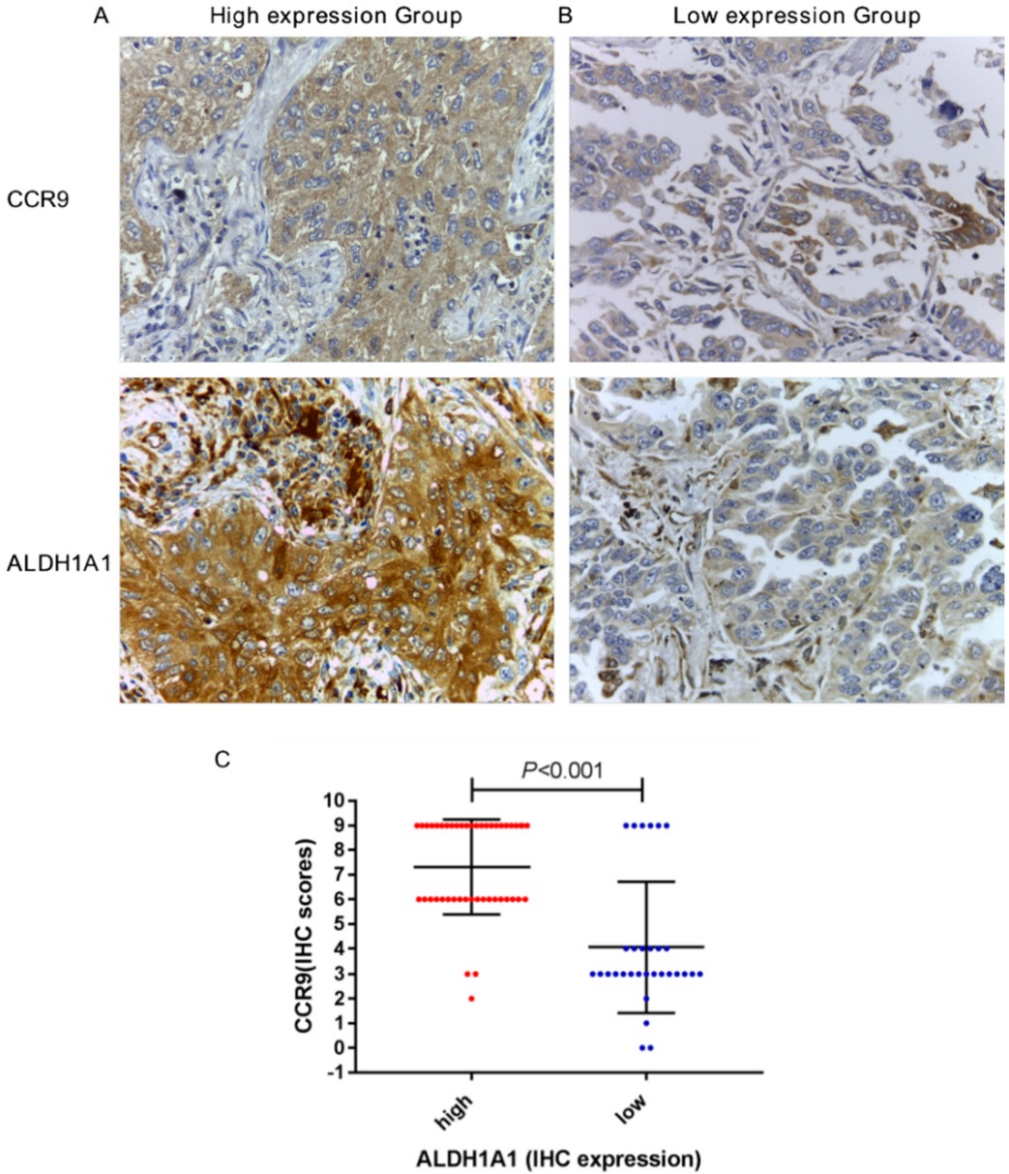
** CCR9 expression was positively correlated with ALDH1A1^+^CSCS expression in lung adenocarcinoma**. Representative microphotographs of CCR9 and ALDH1A1 expression in the lung adenocarcinoma patients: (A) The same patient with both high CCR9 expression and high ALDH1A1 expression; (B) The same patient with both low CCR9 expression and low ALDH1A1 expression. (C) There was a positive correlation between the expression of CCR9 and ALDH1A1^+^ CSCs. Original magnification: 400×.

We further studied the correlation between the expression of CCR9 and ALDH1A. CCR9 and ALDH1A1 were both highly expressed in 42 cases and both lowly expressed in 25 cases. Figure 2A and B showed the representative microphotographs from the same patients (high expression and low expression). There was positive correlation between CCR9 expression and ALDH1A1 expression, with a correlation coefficient of 0.602 (*P* < 0.001, Figure 2C). As shown in Figure 2C, the immunostaining scores of CCR9 in ALDH1A1 high expression group was generally higher than that of in ALDH1A1 low expression group.

### CCR9 was highly expressed on ALDH^high^ cancer stem cells (CSCs)

**Figure 3:**
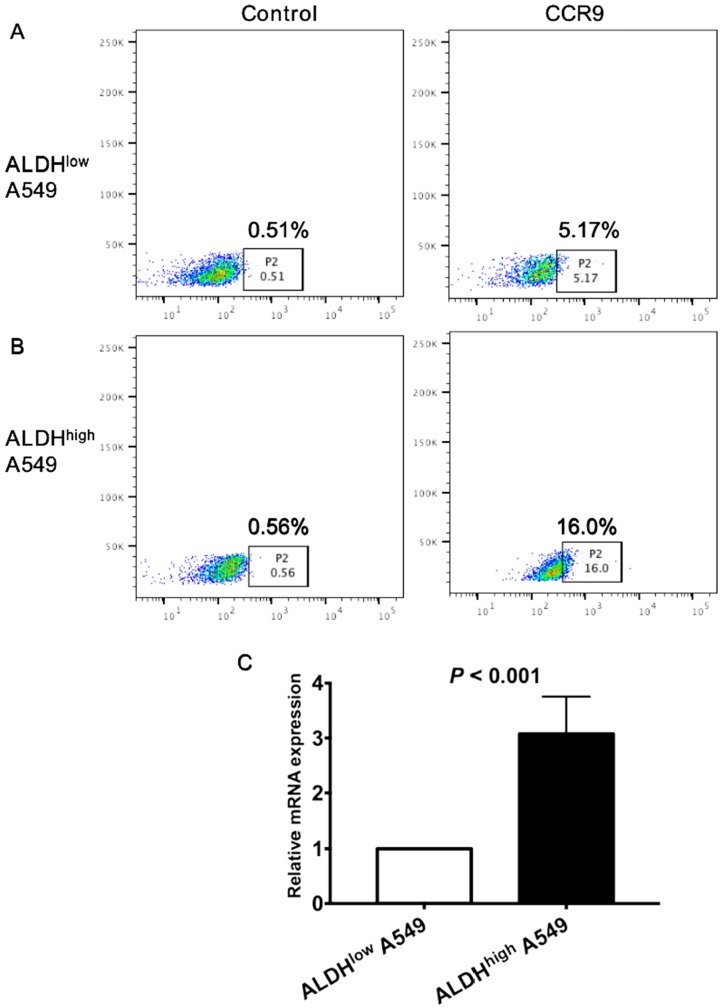
** The expression CCR9 on ALDH^high^ CSCs was much higher than that on ALDH^low^ tumor cells.** The expression of CCR9 on ALDH^high^ CSCs (A) and ALDH^low^ non- CSCs (B) was detected using flow cytometry. (C) The expression of CCR9 on ALDH^low^ cells and ALDH^high^ cells were analyzed using real time quantitative PCR.

To further clarify the correlation between CCR9 and CSCs, we detected the expression of CCR9 on CSCs. Both ALDH^high^ CSCs and ALDH^low^ non-CSCs were isolated from lung adenocarcinoma cell line A549 using ALDEFLUOR assay. Expression level of CCR9 on ALDH^high^ CSCs was much higher than that on ALDH^low^ cells. Representative graphs of flow cytometry were demonstrated in Figure 3A and B. The result was further confirmed by real time quantitative PCR. ALDH^high^ A549 cells expressed more CCR9 than ALDH^low^ cells (Figure 3C, *P* < 0.001).

### CCR9/CCL25 promoted the migration and invasion of lung adenocarcinoma cancer stem cells (CSCs)

**Figure 4:**
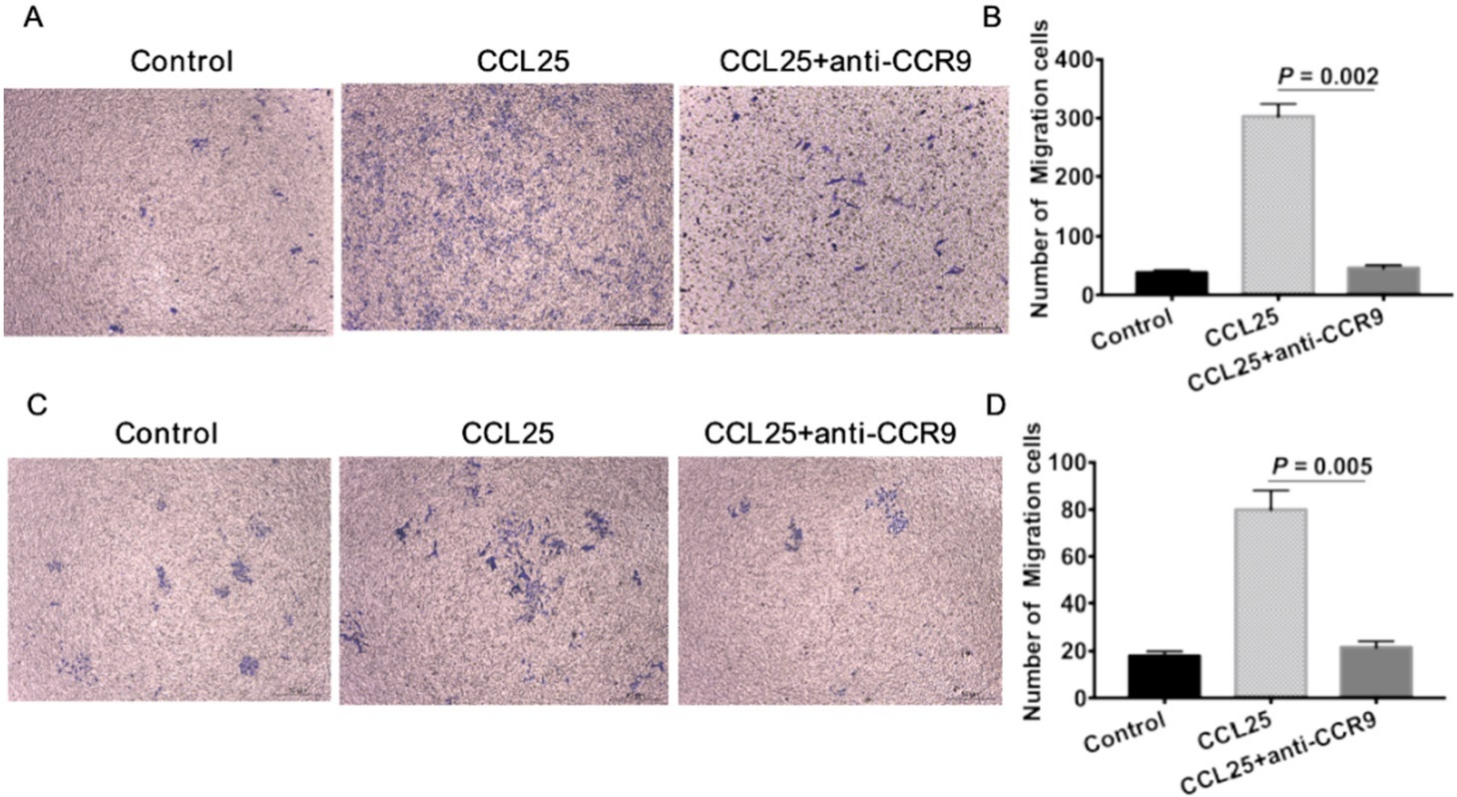
** CCR9/CCL25 promoted migration and invasion of ALDH^+^ cancer stem cells.** ALDH^high^ CSCs were isolated using ALDEFLUOR kit. CSCs showed increased migration (A) and invasion (B) capabilities towards CCL25 stimulation. Additional application of CCR9 antibody inhibited CCL25-mediated cell migration (A) and invasion (B). Representative images are shown on the left, and the quantification of the results are shown on the right. Original magnification: 40×.

To further verify the effect of CCR9/CCL25 on CSCs, we firstly isolated ALDH^high^ CSCs using flow cytometry as previously described 22, 23. The numbers of A549 cells migrated in response to CCL25 were much more than the untreated tumor cells (Figure 4A, B). CCL25-induced cell migration was inhibited by additional application of anti-CCR9 antibody (Figure 4A, B). Consistent with the migration assay, there were much more tumor cells invaded to the lower chamber in response to CCL25 stimulation (Figure 4C, D). And the invasive capability of tumor cells in response to CCL25 was inhibited by CCR9 neutralization (Figure 4C, D).

## Discussion

Chemokines/chemokine receptors take part in all stages of tumor development, including immune cells recruitment, neovascularization, tumor proliferation, invasion, and metastasis 26, 27. CCR9 interacts with its exclusive ligand CCL25 and results in trafficking of both lymphocytes and tumor cells to participate in tumor immunity and tumor development 11, 16, 17, 27-30. More and more studies have indicated that CCR9 could be viewed as an oncogenic biomarker in predicting metastasis and prognosis for cancer patients. Increased expression of CCR9 and CCL25 have been identified in various tumors, such as nasopharyngeal carcinoma and ovarian cancer 9, 31. Upregulated expression of CCR9 and CCL25 were correlated with advanced tumor stage and poor overall survival 9, 31. Consistantly, our study also demonstrated that increased CCR9 expression was detected in lung adenocarcinoma tissues. High expression of CCR9 was correlated with distant metastasis and poor overall survival in lung adenocarcinoma patients. However, the expression of CCL25 was barely detect in lung adenocarcinoma tissues. Of note, Gupta et al failed to detect the expression of CCL25 in lung cancer cells. Instead, they detected the higher serum concentration of CCL25 in lung adenocarcinoma patients than that in the lung squamous cell carcinoma patients and healthy controls 17.

Prostate cancer cells highly expressing CCR9 could promote tumor cell migration and invasion 11. Expression of CCR9 mediated melanoma specific metastasis to the small intestine 30, 32. The expression of CCR9 also reduced the sensitivity of tumor cells to chemotherapy, which promoted cancer cells invasion and metastasis 29. Chen et al engineered colorectal cancer cells to express CCR9, resulting in the promotion of distant metastasis 33. It's generally accepted that the existence of cancer stem cells (CSCs) in the tumor residue contributing to chemo and radio-resistance, tumor invasion, and metastasis 34, 35. CCR9 was correlated with chemo-resistance, tumor invasion and metastasis, which indicating that there might be cross-talk between CCR9 and cancer stem cells in the tumor microenvironment.

CCR9/CCL25 has been reported to play important role in attracting stem cells 36. However, there were little studies concerning the effect of CCR9/CCL25 signal on cancer stem cells. CCR9 was detected on circulating tumor cells of melanoma patients, along with tumor cell migration and distant metastasis 37. Zhang et al found that CCL25 stimulation up-regulated the expression of epithelial-mesenchymal transition (EMT) markers in breast cancer cells and hepatocellular carcinoma cells^38^. Chen et al detected the expression of CCR9 in colon-cancer-initiating cell lines generated from the early stage of colorectal cancer, which led to the formation of xenograft tumors^39^. Thus, we further investigated the correlation between CCR9 and CSCs. The immunohistochemistry using serial paraffin- embedded slides demonstrated that increased expression of ALDH1A1+CSCs was associated with distant metastasis and poor prognosis of lung adenocarcinoma patients. Besides, the expression of CCR9 was positively correlated with ALDH1A1 expression in the same tumor microenvironment. The expression of CCR9 was enhanced on ALDH^high^ CSCs. Additional supplement of CCL25 promoted the migration and invasion of ALDH^high^ CSCs, which was reversed by the application of CCR9 neutralizing antibody.

In conclusion, our study found that there was positive correlation between CCR9 and ALDH1A1+ CSCs. Increased expression of CCR9 was detected on CSCs, which resulted in the migration and invasion of cancer stem cells through CCR9/CCL25 axis. This might contribute to the distant metastasis of lung adenocarcinoma. This study will provide more evidence for us to further study the application of CCR9 as an effective and novel strategy in inhibiting the metastasis of lung adenocarcinoma.

## Supplementary Material

Supplementary figure.

## Figures and Tables

**Table 1:** The relationship between CCR9 expression and distant metastasis in lung adenocarcinoma

Distant metastasis	n	CCR9		Χ^2^	*P-*value
		High expression	Low expression		
M0	52	28(53.8%)	24(46.2%)	4.934	0.026*
M1	24	20(83.3%)	4(16.7%)		

**Table 2:** The relationship between ALDH1A1^+^CSCs expression and distant metastasis in lung adenocarcinoma

Distant metastasis	n	ALDH1A1		Χ^2^	*P-*value
		High expression	Low expression		
M0	52	26(50.0%)	26(50.0%)	5.784	0.016*
M1	24	19(79.2%)	5(20.8%)		

## Abbreviations

CCR9: CC chemokine receptor 9
ALDH1A1: aldehyde dehydrogenase 1 family member A1
CSCs: cancer stem cells
NSCLC: Non-small cell lung cancer
RT-qPCR: real time quantitative PCR
GAPDH: glyceraldehyde-3-phosphate dehydrogenase
DEAB: diethylaminobenzaldehyde
OS: overall survival
EMT: epithelial-mesenchymal transition
