# Supplementary material for: CCR9 Promotes Migration and Invasion of Lung Adenocarcinoma Cancer Stem Cells
Source: Int J Med Sci. 2020 Mar 26;17(7):912–20. doi: 10.7150/ijms.40864 (PMC7163367; doi:10.7150/ijms.40864)
Supplement: Supplementary file 1 — Supplementary figure. [file ijmsv17p0912s1.pdf]

Supplementary Figure 1. The expression of CCL25 in lung adenocarcinoma. Immunohistochemistry was performed to detect the expression of CCL25 in lung adenocarcinoma tissues. (A) and (B) demonstrate negative CCL25 expression in two samples. Original magnification: 400×.

A B

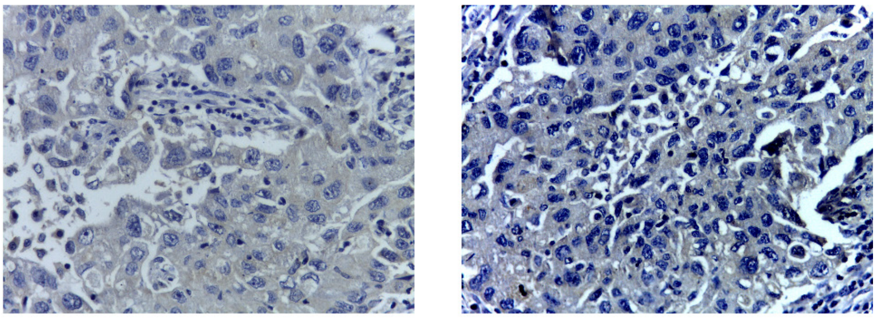

Supplementary Figure 1
